# Non-imidazole histamine H_3_ ligands: part V. synthesis and preliminary pharmacological investigation of 1-[2-thiazol-4-yl- and 1-[2-thiazol-5-yl-(2-aminoethyl)]-4-*n*-propylpiperazine derivatives

**DOI:** 10.1007/s00044-012-0372-8

**Published:** 2012-11-29

**Authors:** Roman Guryn, Marek Staszewski, Krzysztof Walczyński

**Affiliations:** Department of Synthesis and Technology of Drugs, Medical University, Muszyńskiego Street 1, 90-145 Łódź, Poland

**Keywords:** Histamine H_3_-receptor, H_3_-antagonists, 1-[2-thiazol-4-yl-(2-aminoethyl)]- and 1-[2-thiazol-5-yl-(2-aminoethyl)]-4-*n*-propylpiperazine derivatives

## Abstract

Series of 1-[2-thiazol-4-yl-(2-aminoethyl)]- and 1-[2-thiazol-5-yl-(2-aminoethyl)]-4-*n*-propylpiperazine derivatives have been prepared and in vitro tested as H_3_-receptor antagonists (the electrically evoked contraction of the guinea-pig jejunum). It appeared that by comparison of homologous pairs, the 1-[2-thiazol-5-yl-(2-aminoethyl)]-4-*n*-propylpiperazines (**3a**,**b** and **4a**–**d**) have much higher potency than their analogous 1-[2-thiazol-4-yl-(2-aminoethyl)]-4-*n*-propylpiperazines (**2a**–**k**). Based on the obtained results, we observed the 5-position of 2-methyl-2-R-aminoethyl substituents in the thiazole ring is favourable for histamine H_3_ receptor antagonist activity, whereas its presence in position 4 leads, almost in each case, to strong decrease of activity.

## Introduction

Histamine plays a variety of physiological roles in the central nervous system (CNS) and peripheral tissues through the four known G protein-coupled receptors, H_1_, H_2_, H_3_ and H_4_ (Hough, 2001). H_1_ and H_2_ receptor antagonists are well-known therapeutic agents and are in use for the treatment of allergic disease (Leurs *et* *al*., 2002) and peptic ulcer (Brimblecombe *et* *al*., 1978), respectively. The newly discovered H_4_ receptor seems to have a role in regulating inflammatory responses (Thurmond *et* *al*., 2004). The histamine H_3_ receptor, which was discovered in 1983 by Arrang and co-workers (Arrang *et* *al*., 1983), mainly located in the CNS, is a presynaptic autoreceptor that does not only modulate the production and the release of histamine from histaminergic neurons (Arrang *et* *al*., 1987) but also regulates the release of other neurotransmitters like acetylocholine (Clapham and Kilpatrick, 1992; Yokatoni *et* *al*., 2000), dopamine (Schlicker *et* *al*., 1993), norepinephrine (Schlicker *et* *al*., 1990), serotonin (Schlicker *et* *al*., 1988) and glutamate (Brown and Reymann, 1996) in both the CNS and peripheral nervous system. Enhancement of neurotransmitter release by histamine H_3_ receptor antagonist shows a clinical approach to the treatment of several CNS disorders (Esbenshade *et* *al*., 2006; Cemkov *et* *al*., 2009), including attention deficit hyperactivity disorder (Quades, 1987), sleep disorders (Monti, 1993), epilepsy (Vahora *et* *al*., 2001) and schizophrenia (Velligan and Miller, 1999). Pharmacological data also suggest a potential role for H_3_ antagonists in the control of feeding, appetite, and support the role of H_3_ receptor in obesity (Hancock, 2003; Hancock *et* *al*., 2004).

**Chart 1:**
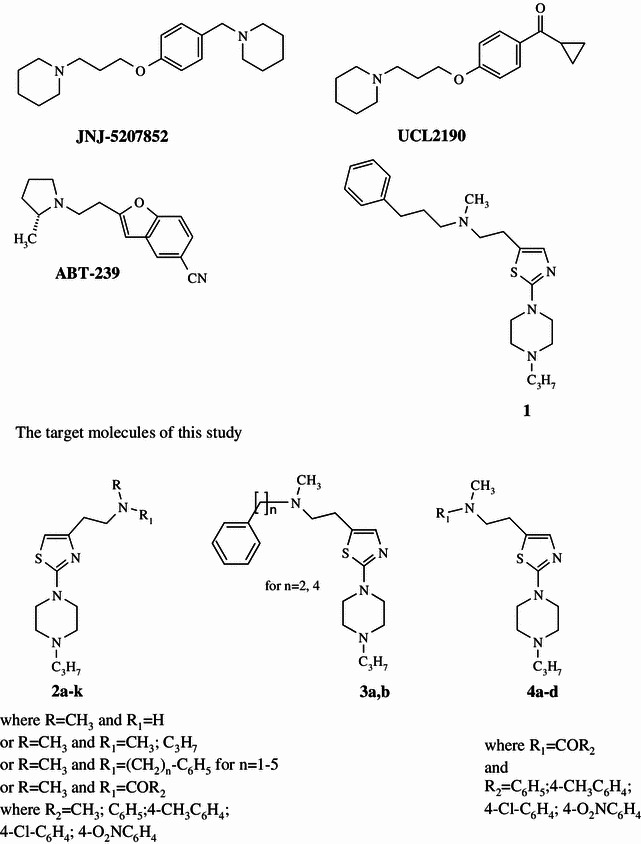
Representative non-imidazole H_3_-histamine receptor antagonists and the target molecules of this study

Early generation of H_3_ receptor ligands were based on structures containing the imidazole moiety, many of which have found utility as pharmacological tools (Stark *et* *al*., 1996; Van der Goot and Timmerman, 2000). However, antagonist carrying on the imidazole heterocycle is the potential issue for drug–drug interactions through inhibition of hepatic cytochrome P_450_ enzymes and poor CNS penetration (Lin and Lu, 1998; Zhang *et* *al*., 2005). For these reasons, and after the successful cloning of the human histamine H_3_ receptor by Lovenberg (Lovenberg *et* *al*., 1999), efforts have been directed towards the discovery of H_3_ antagonists without an imidazole moiety as these compounds may offer improvements in binding affinity, CNS penetration, and reduced potential for cytochrome P_450_ enzymes inhibition (Cowart *et* *al*., 2004). A number of non-imidazole antagonists have since been reported (Ganellin *et* *al*., 1998; Celanire *et* *al*., 2005). Representative examples of non-imidazole H_3_ antagonists included among others were JNJ-5207852 (hH_3_RK_i_ = 0.6 nM) (Apodaca *et* *al*., 2003), UCL 2190 (rH_3_RK_i_ = 4 nM) (Meier *et* *al*., 2001) and ABT-239 (hH_3_RK_i_ = 0.45 nM) (Cowart *et* *al*., 2002) (Chart 1).

Previously, our laboratory has described several non-imidazole piperazine-based histamine H_3_ antagonists, consisting of 1-(2-thiazolobenzo)-, 1-(2-thiazolopyridine)- and 1-[2-thiazol-5-yl-(2-aminoethyl)] moieties with moderate to pronounced affinity for the receptor (Walczyński *et* *al*., 1999, 2005; Frymarkiewicz and Walczynski, 2009). The SAR of 1-[(2-thiazolobenzo)-4-*n*-propyl]piperazines and 1-[(2-thiazolopyridine)-4-*n*-propyl]piperazines series, showed no significant difference in H_3_ activities (Walczyński *et* *al*., 1999, 2005). These results prompted us to replace the benzo ring by 2-methyl-2-alkylaminoethyl amide, 2-methyl-2-alkylaminoethyl and 2-methyl-2-phenylalkylaminoethyl chains at position 5 of 1-(2-thiazol-5-yl)-4-*n*-propylpiperazine moiety. The highest affinity for these series has been seen in the compound with the *N*-methyl-*N*-phenylpropylamino substituent **1** (Chart 1; pA_2_ = 8.27; electric field stimulation assay on guinea-pig jejunum) and with slightly lower potencies for compounds carrying on *N*-methyl*-N*-benzylamino and *N*,*N*-dimethylamino substituents with pA_2_ = 7.75 and 7.78, respectively (Frymarkiewicz and Walczynski, 2009).

In continuation of our earlier work, we studied the influence, on H_3_-receptor antagonistic activity, of the introduction of 2-CH_3_-2-R-aminoethyl-substitution at position 4 of the thiazole ring. Therefore, the series of 1-[2-thiazol-4-yl-(2-aminoethyl)]-4-*n*-propylpiperazines **2a**–**k** (Chart 1), bearing the substituents showing the highest affinity in previously described 1-[2-thiazol-5-yl-(2-aminoethyl)]-4-*n*-propylpiperazines (Frymarkiewicz and Walczynski, 2009), was prepared and pharmacologically evaluated (electric field stimulation assay on guinea-pig jejunum). In addition, with the aim of the complement 1-[2-thiazol-5-yl-(2-aminoethyl)]-4-*n*-propylpiperazines series, 1-[2-thiazol-5-yl-(2-methyl-2-phenylethyl)]- **3a**, 1-[2-thiazol-5-yl-(2-methyl-2-phenylbutylaminoethyl)]-4-*n*-propylpiperazine **3b** and 1-[2-thiazol-5-yl-(2-methyl-2-phenylcarbonylaminoethyl)]-4-*n*-propylpiperazine amides **4a**–**d** (Chart 1) were synthesized.

In this study, we report on synthesis and preliminary pharmacological investigation of new 1-[2-thiazol-5-yl-(2-aminoethyl)]-4-*n*-propylpiperazine derivatives **2** and 1-[2-thiazol-5-yl-(2-methyl-2-phenylethyl-, 1-[2-thiazol-5-yl-(2-methyl-2-phenylbutylaminoethyl)]-4-*n*-propylpiperazines **3** and 1-[2-thiazol-5-yl-(2-methyl-2-phenylcarbonylaminoethyl)]-4-*n*-propylpiperazine amides **4**.

## Chemistry

**Scheme 1:**
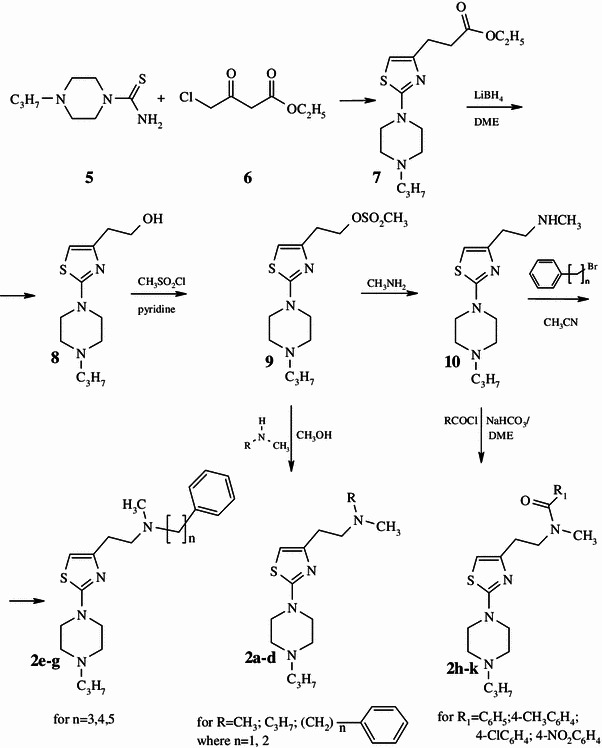
Synthesis of 1-[2-thiazol-4-yl-(2-aminoethyl)]-4-*n*-propylpiperazines **2a**–**k**

**Scheme 2:**
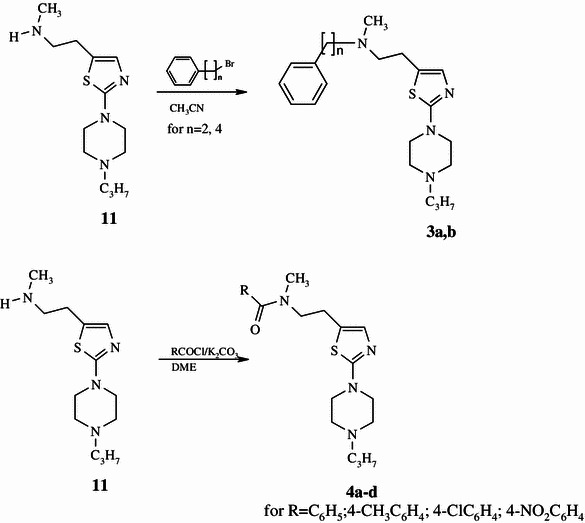
Synthesis of 1-[2-thiazol-5-yl-(2-methyl-2-phenylalkylaminoethyl)]-4-*n*-propyl- piperazines 3**a**, **b** and 1-[2-thiazol-5-yl-(2-methyl-2-phenylcarbonylaminoethyl)]-4-*n*-propyl- piperazine amides **4a**–**d**

The general synthetic procedure used in this study is illustrated in Schemes 1 and 2. 1-[2-Thiazol-4-yl-(2-methylaminoethyl)]-4-*n*-propylpiperazine **10** (Scheme 1) was prepared from compound **5** by four-step synthesis including cyclization reaction of 1-(4-*n*-propyl)piperazine thioamide **5** with ethyl 4-chloroacetoacetate **6** to 1-[2-thiazol-4-yl-(2-methoxycarbonylethyl)]-4-*n*-propylpiperazine **7**, reduction with LiAlH_4_ in dry ethyl ether to 1-[2-thiazol-4-yl-(2-hydroxyethyl)]-4-*n*-propylpiperazine **8**, mesylation with methanesulfonyl chloride in dry pyridine to 1-[2-thiazol-4-yl-(2-mesyloxyethyl)]-4-*n*-propylpiperazine **9** and finally through nucleophilic displacement of the mesyloxy group by methylamine in methanol to 1-[2-thiazol-4-yl-(2-methylaminoethyl)]-4-*n*-propylpiperazine **10**. 1-[2-Thiazol-4-yl-(2-methy-2-alkylaminoethyl)]-4-*n*-propylpiperazines **2a**,**b** and 1-[2-thiazol-4-yl-(2-methy-2-phenylalkylaminoethyl)]-4-*n*-propylpiperazines **2c**,**d** were prepared from 1-[2-thiazol-4-yl-(2-mesyloxyethyl)]-4-*n*-propylpiperazine **9** through nucleophilic substitution of the mesyloxy group by an appropriate secondary amine in methanol. Compounds **2e**–**g**, 1-[2-thiazol-4-yl-(2-methyl-2-phenylalkylaminoethyl)]-4-*n*-propylpiperazine, were obtained from 1-[2-thiazol-4-yl-(2-methylaminoethyl)]-4-*n*-propylpiperazine **10** by alkylation with the corresponding primary phenyloalkyl halides in acetonitrile followed by purification with column chromatography. [2-Thiazol-4-yl-(2-metyl-2-phenylcarbonylaminoethyl)]-4-*n*-propylpiperazine amides **2h**–**k** were obtained by standard methods. Compound **10** was acetylated with an appropriate acid chloride in the presence of NaHCO_3_ in DME, followed by purification with column chromatography.

Compounds **3a**,** b**, 1-[2-thiazol-5-yl-(2-methyl-2-phenylalkylaminoethyl)]-4-*n*-propylpiperazine (Scheme 2), were synthesized from compound **11** by alkylation with the corresponding primary phenylalkyl halides in acetonitrile followed by purification with column chromatography. Amides **4a**–**d** were obtained by acetylation of 1-[2-thiazol-5-yl-(2-methylaminoethyl)]-4-*n*-propylpiperazine **11** (Scheme 2) with an appropriate acid chloride with the presence of K_2_CO_3_ in DME, followed by purification with column chromatography.

All free bases were dissolved in small amount of *n*-propanol and treated with methanolic HBr. The hydrobromides crystallized as white solid.

The 1-(4-*n*-propyl)piperazine thioamide (**5**) was directly obtained by the reaction of the 1-*n*-propylpiperazine dihydrobromide with potassium thiocyanate in aqueous solution (Frymarkiewicz and Walczynski, 2009).

The 5-phenylpentyl bromide was obtained according to Collins (Collins and Davis, 1961). The 5-phenyl-1-pentanol was converted into the bromide by treatment with 50 % aqueous hydrobromic acid and concentrated sulphuric acid.

The ethyl 4-chloroacetoacetate, 1-*n*-propylpiperazine dihydrobromide, benzyl bromide, 1-bromo-3-phenylpropane, 1-bromo-4-phenylbutane 5-phenyl-1-pentanol, dimethylamine solution in methanol, *N*-methylpropylamine, *N*-benzylmethylamine, *N*-methyl-2-phenethylamine, benzoyl chloride, *p*-toluoyl chloride, 4-chlorobenzoyl chloride and 4-nitrobenzoyl chloride were all purchased from commercial sources.

## Results and discussion

The compounds were in vitro tested as H_3_ receptor antagonists—the electrically evoked contraction of the guinea-pig jejunum.

The presented series of 1-[2-thiazol-4-yl-(2-aminoethyl)]-4-*n*-propylpiperazines (**2a**–**k**) and their analogous 1-[2-thiazol-5-yl-(2-aminoethyl)]-4-*n*-propylpiperazine (**3a**,**b** and **4a**–**d**) derivatives possess weak to pronounced H_3_-receptor antagonist potency (Table 1).

**Table 1:** H_3_ antagonistic activity of 1-[2-thiazol-4-yl-(2-aminoethyl)]-4-*n*-propylpiperazines **2a**–**k** and their homologous series 1-[2-thiazol-5-yl-(2-aminoethyl)]-4-*n*-propylpiperazines **3a**,**b** and **4a**–**d** as tested on the in vitro test system on the guinea-pig jejunum

*R*	Cpd.	*n*	pA_2_ (sem) H_3_	*N* (caviae)	Cpd.	*m*	pA_2_ (sem) H_3_	*N* (caviae)
CH_3_–	**2a**	3	6.76 (014)	9 (3)	*	3	7.78 (0.03)	21 (6)
C_3_H_7_–	**2b**	3	6.92 (0.10)	9 (3)	*	3	7.53 (0.05)	18 (5)
Ph–CH_2_–	**2c**	3	7.12 (0.18)	9 (3)	*	3	7.76 (0.06)	18 (5)
Ph–(CH_2_)_2_–	**2d**	3	6.81 (0.15)	9 (3)	**3a**	3	7.61 (0.06)	9 (3)
Ph–(CH_2_)_3_–	**2e**	3	6.61 (0.11)	9 (3)	*	3	8.27 (0.05)	20 (6)
Ph–(CH_2_)_4_–	**2f**	3	6.72 (0.11)	9 (3)	**3b**	3	7.80 (0.03)	9 (3)
Ph–(CH_2_)_5_–	**2g**	3	6.69 (0.05)	9 (3)	*	3	7.25 (0.04)	11 (5)
Ph–CO–	**2h**	2	5.65 (0.00)	6 (2)	**4a**	2	7.45 (0.01)	9 (3)
*p*-CH_3_–Ph–CO–	**2i**	2	5.80 (0.10)	9 (3)	**4b**	2	7.61 (0.16)	9 (3)
*p*-Cl–Ph–CO–	**2j**	2	6.23 (0.11)	9 (3)	**4c**	2	7.73 (0.11)	9 (3)
*p*-NO_2_–Ph–CO–	**2k**	2	6.03 (0.02)	9 (3)	**4d**	2	7.76 (0.02)	9 (3)

Thioperamide—pA_2_ H_3_ = 8.43, (sem) (0.07); *N* (caviae)—18 (6)

H_3_ antagonistic activity of all compounds marked with asterisk was described in previous paper (Frymarkiewicz and Walczynski, 2009)

*sem* standard error of the mean, *N* number of different animal preparation; *cavie* number of animals; *m* and *n* number of HBr

The introduction of 2-methyl-2-R-aminoethyl-substituents at position 4 of the thiazole ring led to the derivatives **2a**, **b**, **d**–**k** having, independent of the sort of substituent, weak activity, except for derivative **2c** showing moderate affinity with pA_2_ = 7.12.

It appeared that by comparison of homologous pairs, the 1-[2-thiazol-5-yl-(2-aminoethyl)]-4-*n*-propylpiperazines (**3a**,**b** and **4a**–**d**) have much higher potency than their analogous 1-[2-thiazol-4-yl-(2-aminoethyl)]-4-*n*-propylpiperazines (**2a**–**k**). The differences are observed inside of each series. In the case of 1-[2-thiazol-4-yl-(2-aminoethyl)]-4-*n*-propylpiperazines, elongation of alkyl chain from one to three methylene groups results in an increase of potency for **2a** pA_2_ = 6.76 and **2b** pA_2_ = 6.96, this is in opposition to the 1-[2-thiazol-5-yl-(2-aminoethyl)]-4-*n*-propylpiperazine derivatives where the 1-[2-thiazol-5-yl-(2-*N*,*N*-dimethylaminoethyl)]-4-*n*-propylpiperazine shows slightly higher potency than its *N*-methyl-*N*-propyl analogue (pA_2_ = 7.78; pA_2_ = 7.53, respectively).

In the 2-methyl-*2*-phenylalkyl derivatives of 1-[2-thiazol-4-yl-(2-aminoethyl)]-4-*n*-propylpiperazine (**2c**–**g**), there is no significant difference in affinity. Elongation of alkyl chain from one to five methylene groups does not influence antagonistic activity (pA_2_ ranging from 6.81 for compound **2d** to 6.69 for compound **2g**). In the analogues series, there is no significant difference in affinity among the methyl and ethyl derivatives (pA_2_ = 7.76 and 7.61 for compound **3a**). A further elongation in the alkyl chain length to 3 methylene groups results in an increase of antagonistic activity, reaching the maximum for 1-[2-thiazol-5-yl-(2-methyl-2-phenylpropylaminoethyl)]-4-*n*-propylpiperazine (pA_2_ = 8.27); activity decreases on further lengthening up to 5 methylene groups (pA_2_ = 7.80 for compound **3b** and 7.25 for 1-[2-thiazol-5-yl-(2-phenylpentylmethylaminoethyl)]-4-*n*-propylpiperazine). Replacement of hydrogen by *p*-benzoyl substituent at the end of *N*-methyl group leads to the compounds **2h**–**k** (pA_2_ from 5.65 to 6.23) and their analogues **4a**–**d** (pA_2_ from 7.45 to 7.76). By comparison of homologous pairs, the 1-[2-thiazol-5-yl-(2-methyl-2-phenylcarbonylaminoethyl)]-4-*n*-propylpiperazine amides **4a**–**d** have much higher potency than their analogous 1-[2-thiazol-4-yl-(2-methyl-2-phenylcarbonylaminoethyl)]-4-*n*-propylpiperazine amides **2h**–**k**. In both series, a slightly higher activity is observed for compounds carrying on electron-withdrawing substituent at *para*-position in the benzene ring.

Summarizing, 1-[2-thiazol-5-yl-(2-aminoethyl)]-4-*n*-propylpiperazines display a higher activity than their 1-[2-thiazol-4-yl-(2-aminoethyl)]-4-*n*-propylpiperazine analogues. We observe that the position 5 of 2-methyl-2-R-aminoethyl-substituents in the thiazole ring is favourable for histamine H_3_ receptor antagonist activity, whereas its presence in position 4 leads, almost in each case, to strong decrease of activity.

The highest potency for both homologous series is seen in the compound with the 2-methyl-2-phenylpropylaminoethyl substituent (pA_2_ = 8.27) and with slightly lower potencies for compounds carrying on 2,2-dimethylaminoethyl, 2-methyl-2-(4-chlorophenyl)carbonylaminoethyl and 2-methyl-2-(4-nitrophenyl)-carbonylaminoethyl substituents (pA_2_ = 7.78; pA_2_ = 7.73 and pA_2_ = 7.76, respectively).

## Experimental protocols

General Methods. All melting points (mp) were measured in open capillaries on an electrothermal apparatus and are uncorrected. For all compounds, ^1^H NMR spectra were recorded on a Varian Mercury 300 MHz spectrometer. Chemical shifts are expressed in ppm downfield from internal TMS as reference. ^1^H NMR data are reported in order: multiplicity (br, broad; s, singlet; d, doublet; t, triplet; m, multiplet; * exchangeable by D_2_O) number of protons, and approximate coupling constant in Hertz. ^13^C NMR spectra were recorded on Bruker Avance III 600 MHz spectrometer. Elemental analysis (C, H, N) for all compounds were measured on Perkin Elmer Series II CHNS/O Analyzer 2400 and are within ±0.4 % of the theoretical values. TLC was performed on silica gel 60 F_254_ plates (Merck). Flash column chromatography was carried out using silica gel 60 Å  50 μm (J. T. Baker B. V.), employing the same eluent as was indicated by TLC.

### Chemistry

#### The synthesis of 1-[2-thiazol-4-yl-(2-methoxycarbonylethyl)]-4-*n*-propylpiperazine (**7**)

The 1-(4-*n*-propyl)piperazine thioamide (**5**) (0.032 mol) was added to a solution of ethyl 4-chloroacetoacetate (**6**) (0.032 mol) in 70 mL of *n*-propanol. The reaction mixture was heated at 90 °C for 6 h. After cooling, the solvent was removed in vacuo. The hydrochloride product was obtained as brown solid. The free base was obtained as follows: the hydrochloride of the 1-[2-thiazol-4-yl-(2-methoxycarbonylethyl)]-4-*n*-propylpiperazine (**7**) was mixed with saturated aqueous sodium bicarbonate solution for 1 h at room temperature and then water layer was extracted with dichloromethane (2 × 30 mL). The organic extracts were washed with water (3 × 30 mL), dried (Na_2_SO_4_), filtered and evaporated to give compound **7** as a sticky oil: The free base was dissolved in small amount of *n*-propanol and treated with methanolic HBr. The dihydrobromide crystallized as white solid.

**7**. C_14_H_23_N_3_O_2_S (*M* = 297); yield 82.6 %; sticky oil; ^1^H NMR (CDCl_3_) δ: 0.89–0.95 (t, 3H, CH_2_ CH_3_ J = 7.5 Hz); 1.25–1.29(t, 3H, CH_3_CH_2_O–) 1.48–1.60 (m, 2H, –CH_2_ CH_2_ CH_3_); *2*.33–2.38 (m, 2H, –CH_3_CH_2_ CH_2_–); 2.52–2.56 (m, 4H CH_2_ CH_2_N); 3.46–3.50 (m, 4H, –CH_2_ CH_2_N); 3.60 (s, 2H, CH_2_CO–) 4.14–4.22(q, 2H CH_2_O, J = 7.2 Hz) 6,39 (s, 1H, H_thiazole_); TLC (methylene chloride:methanol 19:1) R_f_ = 0.21

**Table:** Elemental analysis for dihydrobromide C_14_H_25_Br_2_N_3_O_2_ S (459.26)

	C	H	N
Calculated	36.61 %	5.49 %	9.15 %
Found	36.25 %	5.38 %	9.18 %

mp_dihydrobromide_ 220–222 °C

#### The synthesis of 1-[2-thiazol-4-yl-(2-hydroxyethyl)]-4-*n*-propylpiperazine (**8**)

To a solution of the 1-[2-thiazol-4-yl-(2-methoxycarbonylethyl)]-4-*n*-propylpiperazine (**7**) (0.032 mol) in 110 mL of DME at 55 °C, LiBH_4_ (0.055 mol) was added. The mixture was stirred at 70 °C for 24 h. The solvent was evaporated and remaining material was dissolved in 60 mL of methanol and was heated at 70 °C for 24 h. The solvent was evaporated and the residue was purified by column chromatography on silica gel. The title products were obtained as sticky oil. The free base was dissolved in small amount of *n*-propanol and treated with methanolic HBr. The dihydrobromide crystallized as white solid.

**8**. C_12_H_21_N_3_OS (*M* = 256); yield 75.0 %.; ^1^H NMR (CDCl_3_) δ: 0.89–0.95 (t, 3H, CH_2_ CH_3_ J = 7.5 Hz); 1.51–1.60 (m, 2H, –CH_2_ CH_2_ CH_3_); 2.33–2.38 (m, 2H, –CH_3_CH_2_ CH_2_–); 2.52–2.56 (m, 4H CH_2_ CH_2_N); 2.75–2.78 (t, 2H, CH_2_-thiazole J = 5.7 Hz); 3.45–3.49 (m, 4H, –CH_2_ CH_2_N); 3.84–3.87 (t, 2H CH_2_OH, J = 5.7 Hz) 4.01 (s* br, H, OH–) 6.20 (s, 1H, H_thiazole_); TLC (methylen chloride:methanol 10:1)* R*_f_ = 0.27.

**Table:** Elemental analysis for dihydrobromide C_12_H_21_N_3_OSx2HBr (*M* = 417,22)

	C	H	N
Calculated	34.54 %	5.56 %	10.07 %
Found	34.30 %	5.52 %	10.07 %

mp_dihydrobromide_ 244–246 °C

#### The synthesis of 1-[2-thiazol-4-yl-(2-mesyloxyethyl)]-4-*n*-propylpiperazine (**9**)

To a cooled solution of the 1-[2-thiazol-4-yl-(2-hydroxyethyl)]-4-*n*-propylpiperazine (**8**) (0.009 mol) in 10 mL of dry pyridine, while stirring, methanesulfonyl chloride (0.009 mol) was added dropwise. The mixture was stirred at room temperature for 0.5 h. Then, reaction mixture was poured out in ice-cold water (40 mL) and extracted with ethyl ether (3 × 50 mL). The combined organic extracts were dried (Na_2_SO_4_), filtered and evaporated to give compound **9** as a sticky yellow oil. The crude compound **9** was used in the next step without further purification.

**9**. C_13_H_23_N_3_O_3_S_2_ (*M* = 333); yield 58.1 %; ^1^H NMR (CDCl_3_) δ: 0.90–0.95 (t, 3H, CH_2_ CH_3_ J = 7.4 Hz); 1.48–1.60 (m, 2H, –CH_2_ CH_2_ CH_3_); 2.33–2.38 (m, 2H, –CH_3_CH_2_ CH_2_–); 2.52–2.56 (m, 4H CH_2_ CH_2_N); 2.92 (s, 3H, CH_3_SO_3_) 2.96–3.02 (t, 2H, CH_2_-_thiazole_ J = 6.6 Hz); 3.45–3.48 (m, 4H, –CH_2_ CH_2_N); 4.49–4.52 (t, 2H CH_3_ SO_3_ CH_2_, J = 6.6 Hz) 6,29 (s, 1H, H_thiazole_); TLC (methylen chloride:methanol 10:1) R_f_ = 0.44.

#### *The synthesis of 1*-*[2*-*thiazol*-*4*-*yl*-*(2*-*methylaminoethyl)]*-*4*-*n*-*propylpiperazine* (**10**)

The crude 1-[2-thiazol-4-yl-(2-mesyloxyethyl)]-4-*n*-propylpiperazine **9** (0.008 mol) was dissolved in 30 mL of 40 % solution methylamine in methanol. The mixture was stirred at room temperature for 24 h. Then, organic solvent was evaporated, and residue was dissolved in DME (40 mL), alkalized with solid NaHCO_3_ (0.001 mol) and stirred for 1 h. The mixture was filtered and DME was evaporated to give compound **2** as a yellowish sticky oil. The free base was dissolved in small amount of *n*-propanol and treated with methanolic HBr. The treehydrobromide crystallized as white solid.

**2**. C_13_H_24_N_4_S (*M* = 268); yield 68.9 %; ^1^H NMR (CDCl_3_) δ: 0.90–0.95 (t, 3H, CH_2_ CH_3_ J = 7.5 Hz); 1.50–1.60 (m, 2H, –CH_2_ CH_3_); 2.01 (s* br, 1H, NH); 2.32–2.37 (m, 2H, –CH_3_CH_2_ CH_2_–); 2.45 (s, 3H –CH_3_); 2.52–2.56; (m, 4H CH_2_ CH_2_N); 2.73–2.77 (t, 2H, CH_2_-_thiazole_, J = 6.6 Hz); 2.86–2.91 (t, 2H, CH_2_N J = 6.6 Hz) 3.45–3.48 (m, 4H, CH_2_ CH_2_N); 6.19 (s, 1H, H_thiazole_); TLC (chloroform metanol concentrated ammonium hydroxide 60:10:1) R_f_ = 0.10.

**Table:** Elemental analysis for treehydrobromide C_13_H_27_N_4_ Br_3_S (511,20)

	C	H	N
Calculated	30.54 %	5.32 %	10.96 %
Found	30.61 %	5.23 %	10.97 %

mp_treehydrobromide_ 226–228 °C

#### General method for the preparation of 1-[2-thiazol-4-yl-(2-alkylmethylaminoethyl)] (**2a**,**b**) and 1-[2-thiazol-4-yl-(2-phenylalkylmethylaminoethyl)] 4-*n*-propylpiperazines (**2c**,**d**)

To a solution of 1-[2-thiazol-4-yl-(2-mesyloxyethyl)]-4-*n*-propylpiperazine (**9**) (0.002 mol) in 5.0 mL of methanol, the corresponding amine (0.004 mol) was added (in case of the compound **2a**—33 % solution dimethylamine in methanol was used). The mixture was stirred at 50 °C for 6–10 h. (monitored by TLC). After the completion of reaction, the solvent was evaporated and the residue was alkalized with saturated aqueous NaHCO_3_ solution (15 mL) and stirred for 0.5 h. Then, the mixture was extracted with ethyl ether (3 × 30 mL). The combined organic extracts were dried (Na_2_SO_4_), filtered and evaporated. The residue was purified by column chromatography on silica gel. The title products were obtained as sticky oil. The free base was dissolved in small amount of *n*-propanol and treated with methanolic HBr. The hydrobromide crystallized as white solid to give compounds **2a**–**d**.

**2a**. C_14_H_26_N_4_S (*M* = 282); yield 64.0 %.; ^1^H NMR (CDCl_3_) δ: 0.89–0.94 (t, 3H, –CH_2_ CH_3_ J = 7.2 Hz); 1.47–1.57 (m, 2H, –CH_2_ CH_2_CH_3_); 2.74 (s, 3H, –NCH_3_); *2*.31–2.36 (m, 2H, –CH_3_CH_2_ CH_2_–); 2.51–2.54 (m, 4H CH_2_ CH_2_N); 2.58–2.64 (m, 2H, CH_2_N)); 2.72–2.75 (m, 2H CH_2_-thiazole) 3.45–3.48 (m, 4H, –CH_2_ CH_2_N 6.29 (s, 1H, H_thiazole_); TLC (chloroform:methanol:concentrated ammonium hydroxide 40:10:1) R_f_ = 0.19. mp_threehydrobromide_ 242–244 °C.

IR (for dihydrobromide; KBr) cm^−1^: 3446, 3052, 2962, 2914, 2660, 2587, 2520, 2467, 1613, 1592, 1470, 1432, 1287, 1168, 1133, 997, 969, 813, 662.

**Table:** Elemental analysis for dihydrobromide C_14_H_29_Br_3_N_3_S (525,22)

	C	H	N
Calculated	33.01 %	5.57 %	10.67 %
Found	32.70 %	5.67 %	10.62 %

mp_threehydrobromide_ 242–244 °C

**2b**. C_16_H_30_N_4_S (*M* = 310); yield 68.0 %.; ^1^H NMR (CDCl_3_) δ: 0.87–0.95 (m 6H, –CH_2_ CH_3_ ); 1.47–1.60 (m, 4H, –CH_2_ CH_2_ CH_3_); 2.32 (s, 3H, –NCH_3_); *2*.34–2.43 (m, 4H, –CH_3_CH_2_ CH_2_–); 2.52–2.55 (m, 4H CH_2_ CH_2_N); 2.76 (s, 4H –NCH_2_ CH_2thiazole_); 3.45–3.48 (m, 4H, –CH_2_ CH_2_N); 6.29 (s, 1H, H_thiazole_); TLC (chloroform:methanol:concentrated ammonium hydroxide 40:10:1) R_f_ = 0.25.

IR (for treehydrobromide; KBr) cm^−1^: 3428, 3073, 2963, 2923, 2708, 2655, 2581, 2527, 2469, 1611, 1591, 1459, 1426,1356, 1289, 1239, 1181, 1133, 1099, 1055, 1028, 967, 898, 808, 760, 721, 638, 548.

**Table:** Elemental analysis for treehydrobromide C_16_H_33_Br_3_N_4_S (553.27)

	C	H	N
Calculated	34.73 %	6.01 %	10.13 %
Found	34.71 %	6.07 %	10.13 %

mp_threehydrobromide_ 242–244 °C

**2c**. C_20_H_30_N_4_S (*M* = 359); yield 41.0 %; ^1^H NMR (CDCl_3_) δ: 0.81–0.86 (t 3H, –CH_2_ CH_3_ J = 7.4 Hz); 1.38–1.51 (m, 2H, –CH_2_ CH_2_ CH_3_); 2.16 (s, 3H, –NCH_3_); 2.22–2.28 (m, 4H, –CH_3_CH_2_ CH_2_–); 2.36–2.45 (m, 4H CH_2_ CH_2_N); 2.63–2.76 (m, 4H –NCH_2_ CH_2_-thiazole); 3.35–3.44 (m, 4H, –CH_2_ CH_2_N) 3.46 (s, 2H, CH_2_Ph) 6.29 (s, 1H, H_thiazole_); 7.11–7.26 (m,5H,–H_arom_); TLC (chlorek metylenu:metanol 10:1) R_f_ = 0.23.

IR (for treehydrobromide; KBr) cm^−1^: 3435, 3071, 2963, 2918, 2702, 2653, 2579, 2459, 1615, 1429, 1287, 1185, 1097, 1056, 969, 751, 699.

**Table:** Elemental analysis for treehydrobromide C_20_H_33_Br_3_N_4_S (601.31)

	C	H	N
Calculated	39.95 %	5.53 %	9.32 %
Found	39.57 %	5.47 %	9.19 %

mp_threehydrobromide_ 232–234 °C

**2d**. C_21_H_32_N_4_S (*M* = 373); yield 16.9 %; ^1^H NMR (CDCl_3_) δ: 0.89–0.94 (t 3H, –CH_2_ CH_3_ J = 7.3 Hz); 1.47–1.59 (m, 2H, –CH_2_ CH_2_ CH_3_); 2.32–2.34 (m, 2H, –CH_3_CH_2_ CH_2_–); 2.36 (s, 3H, –NCH_3_); 2.52–2.59 (m, 4H CH_2_ CH_2_N); 2.64–2.70 (m, 2H –NCH_2_ CH_2_-thiazole); 2.70–2.85 (m, 6H, –CH_2_–thiazole –CH_2_ CH_2_Ph,); 3.45–3.54 (m, 4H, –CH_2_ CH_2_N); 6.16 (s, 1H, H_thiazole_); 7.18–7.30 (m, 5H, H_arom_); (TLC (chloroform:metanol:amoniak 60:10:1) R_f_ = 0.55.

IR (for treehydrobromide; KBr) cm^−1^: 3430, 3071, 2962, 2928, 2702, 2653, 2577, 2458, 1613, 1594, 1456, 1411, 1357, 1289, 1181, 1098, 1055, 968, 807, 751, 698.

**Table:** Elemental analysis for treehydrobromide C_21_H_35_Br_3_N_4_S (615.32)

	C	H	N
Calculated	40.72 %	5.70 %	9.05 %
Found	40.57 %	5.37 %	9.02 %

mp_threehydrobromide_ 216–218 °C

#### General method for the preparation of 1-[2-thiazol-4-yl-(2-phenylalkylmethylaminoethyl)] 4-*n*-propylpiperazines (**2e**–**g**) and 1-[2-thiazol-5-yl-(2-phenylalkylmethylaminoethyl)] 4-*n*-propylpiperazines (**3a**,**b**)

To a solution of 1-[2-thiazol-4-yl-(2-methylaminoethyl)]-4-n-propylpiperazine (**10**) (0.002 mol) or 1-[2-thiazol-5-yl-(2-methylaminoethyl)]-4-n-propylpiperazine (**11**) (0.002 mol) with the presence of K_2_CO_3_ (0.003 mol) in 5.0 mL of acetonitrile, the corresponding phenylalkyl bromide (0.002 mol) was added. The mixture was stirred at room temperature for 6–10 h (monitored by TLC). Then, inorganic salt was filtered off and solvent was evaporated. The residue was purified by column chromatography on silica gel. The title products were obtained as sticky oil. The free base was dissolved in small amount of n-propanol and treated with methanolic HBr. The hydrobromide crystallized as white solid to give compounds **2e**–**g** and **3a**,**b**, respectively.

**2e**. C_22_H_34_N_4_S (*M* = 387); yield 39.8 %; ^1^H NMR (CDCl_3_) δ: 0.91–0.96 (t 3H, –CH_2_ CH_3_ J = 7.3 Hz); 1.49–1.62 (m, 2H, –CH_2_ CH_2_ CH_3_); 1.76–1.86 (m, 2H, –CH_2_ CH_2_ CH_2_); 2.29 (s, 3H, –NCH_3_); 2.33–2.38 (m, 2H, –CH_3_CH_2_ CH_2_–); 2.43–2.48 (t, 2H, –NCH_2_CH_2_ CH_2_, J = 7.5 Hz); 2.51–2.63 (m, 6H, –CH_2_CH_2_N, CH_2_Ph,); 2.71(s, 4H, –CH_2_-thiazole CH_2_ CH_2_N); 3.42–3.45 (m, 4H, –CH_2_ CH_2_N); 6.34 (s, 1H, H_thiazole_); 7.12–7.28 (m,5H,–H_arom_);TLC (chloroform:metanol:amoniak 60:10:1) R_f_ = 0.46.

IR (for threehydrobromide; KBr) cm^−1^: 3428, 3075, 2962, 2922, 2649, 2577, 2519, 2458, 2363, 1620, 1453, 1430, 1403, 1286, 1240, 1185, 1134, 1033, 967, 808, 753, 700.

**Table:** Elemental analysis for threehydrobromide C_22_H_37_Br_3_N_4_S (629.7)

	C	H	N
Calculated	41.98 %	5.93 %	8.90 %
Found	41.93 %	5.96 %	8.88 %

mp_threehydrobromide_ 220–222 °C

**2f**. C_23_H_36_N_4_S (*M* = 401); yield 40.5 %; ^1^H NMR (CDCl_3_) δ: 0.90–0.94 (t 3H, –CH_2_ CH_3_ J = 7.3 Hz); 1.47–1.67 (m, 6H, –CH_2_ CH_2_ CH_3_, CH_2_CH_2_N; CH_2_ CH_2_Ph); 2.27 (s, 3H, –NCH_3_); 2.32–2.44 (m, 4H, –CH_3_CH_2_ CH_2_, NCH_2_CH_2_ CH_2_–); 2.41–2.49 (m, 4H CH_2_ CH_2_N); 2.59–2.64 (t, 2H, CH_2_Ph J = 7.2 Hz); 2.72 (s, 4H, –thiazole CH_2_ CH_2_N); 3.42–3.48 (m, 4H, –CH_2_ CH_2_N); 6.16 (s, 1H, H_thiazole_); 7.16–7.29 (m,5H,–H_arom_); TLC (chloroform:metanol:amoniak 60:10:1) R_f_ = 0.49.

IR (for threehydrobromide; KBr) cm^−1^: 3523, 3422, 3067, 2965, 2938, 2705, 2655, 2582, 2529, 2469, 1613, 1592, 1457, 1413, 1357, 1289, 1182, 1097, 1029, 969, 809, 748, 705, 669, 550.

**Table:** Elemental analysis for threehydrobromide C_23_H_39_Br_3_N_4_S (643.7)

	C	H	N
Calculated	42.93 %	6.11 %	8.71 %
Found	42.73 %	6.27 %	8.67 %

mp_threehydrobromide_ 217–219 °C

**2g**. C_24_H_38_N_4_S (*M* = 415); yield 66.8 %; ^1^H NMR (CDCl_3_) δ: 0.88–0.93 (t 3H, –CH_2_ CH_3_ J = 7.3 Hz); 1.27–1.37 (m, 2H, (CH_2_)_2_ CH_2_ (CH_2_)_2_); 1.45–1.65 (m, 6H, –CH_2_ CH_2_ CH_3_, CH_2_CH_2_N); 2.30–2.35 (m, CH_3_CH_2_ CH_2_– NCH_3_); 2.41–2.52 (m, 6H, CH_2_ CH_2_N CH_2_CH_2_Ph 2.56–2.61 (t, 2H –CH_2_Ph 2,76 (s, 4H, thiazole CH_2_ CH_2_N); 3.39–3.46 (m, 4H, –CH_2_ CH_2_N) 6.17 (s, 1H, H_thiazole_); 7.12–7.28 (m,5H,–H_arom_); TLC (chloroform:metanol:amoniak 60:10:1) R_f_ = 0.51.

IR (for threehydrobromide; KBr) cm^−1^: 3427, 3305, 3077, 2937, 2876, 2653, 2580, 2458, 1616, 1597, 1434, 1286, 1185, 1096, 967, 807, 756, 701, 528.

**Table:** Elemental analysis for threehydrobromide C_24_H_41_Br_3_N_4_S (*M* = 657.40)

	C	H	N
Calculated	43.84 %	6.29 %	8.52 %
Found	43.75 %	6.32 %	8.55 %

mp_threehydrobromide_ 214–216 °C

**3a**. C_21_H_32_N_4_S (*M* = 372.56); yield 48.0 %; ^1^H NMR (CDCl_3_) δ: 0.90–0.92 (t 3H. –CH_2_ CH_3_ J = 7.2 Hz); 1.50–1.56 (m, 2H, –CH_2_CH_3_); 2.32–2.34 (m, 2H CH_3_CH_2_ CH_2_N); 2.35 (s, 3H CH_3_N); 2.52–2.53 (m, 4H –CH_2_ CH_2_N); 2.62–2.67 (m, 4H CH_2_Ph CH_2_N) 2.77–2.82 (m, 2H –CH_2_N –CH_2_-tiazol); 3.43–3.45 (m 4H –CH_2_ CH_2_N); 6.87 (s 1H H_thiazole_); 7.16–7.28 (m 5H H_arom._); TLC (chloroform:methanol 9:1) R_f_ = 0.23.

IR (for threehydrobromide; KBr) cm^−1^: 3507, 3451, 3052, 2959, 2915, 2695, 2583, 2526, 1578, 1430, 1409, 1309, 1291, 1243, 1188, 1161, 1093, 1033, 964, 810, 756, 728, 703, 623, 544, 510.

**Table:** Elemental analysis for threehydrobromide C_21_H_35_Br_3_N_4_S (*M* = 615.34)

	C	H	N
Calculated	40.99 %	5.73 %	9.11 %
Found	40.92 %	5.51 %	9.16 %

mp_threehydrobromide_ 204–206 °C

**3b**. C_23_H_36_N_4_S (*M* = 400.62) yield 61.0 %; ^1^H NMR (CDCl_3_) δ: 0.91–0.93 (t, 3H. –CH_2_ CH_3_ J = 7.2 Hz); 1.49–1.56 (m, 4H –CH_2_ CH_2_CH_2_N); 1.62–1.67 (m, 2H CH_2_CH_3_); 2.23 (s, 3H CH_3_N); 2.32–2.34 (m, 2H CH_3_CH_2_ CH_2_N); 2.38–2.40 (t, 2H J = 7.2 Hz CH_2_N); 2.50–2.55 (m, 6H –CH_2_ CH_2_N –CH_2_Ph); 2.61–2.63 (t, 2H J = 7.2 Hz CH_2_N); 2.77–2.79(t, 2H J = 7.2 Hz CH_2_-tiazol); 3.42–3.43 (m, 4H –CH_2_ CH_2_N); 6.87 (s, 1H H_thiazole_); 7.15–7.26 (m 5H H_arom._); TLC (chloroform: methanol 9:1) R_f_ = 0.14.

IR (for threehydrobromide; KBr) cm^−1^: 3471, 3399, 3052, 2938, 2639, 2597, 2473, 1627, 1498, 1434, 1291, 1193, 1027, 964, 846, 752, 722, 597.

**Table:** Elemental analysis for threehydrobromide C_23_H_39_Br_3_N_4_S (*M* = 643.39)

	C	H	N
Calculated	42.93 %	6.11 %	8.71 %
Found	42.87 %	6.14 %	8.78 %

mp_threehydrobromide_ 260–262 °C

#### General method for the preparation of 1-[2-thiazol-4-yl-(2-methyl-2-phenylcarbonylaminoethyl)]-4-*n*-propylpiperazine amides **2h**–**k** and 1-[2-thiazol-5-yl-(2-methyl-2-phenylcarbonylaminoethyl)]-4-*n*-propylpiperazine amides **4a**–**d**

To a solution of 1-[2-thiazol-4-yl-(2-methylaminoethyl)]-4-n-propylpiperazine (**2**) or 1-[2-thiazol-5-yl-(2-methylaminoethyl)]-4-n-propylpiperazine (**11**) (0.001 mol) in 10 mL of DME, the corresponding acid chloride (0.001 mol) was added. After 15 min, NaHCO_3_ (0.001 mol) was added and the mixture was stirred at room temperature for 24 h. The solvent was evaporated and the residue was suspended with H_2_O (30 mL) and extracted with chloroform (3 × 30 mL). The combined organic extracts were dried (Na_2_SO_4_), filtered and evaporated. The residue was purified by column chromatography on silica gel. The title products were obtained as sticky oil. The free base was dissolved in small amount of n-propanol and treated with methanolic HBr. The hydrobromide crystallized as white solid to give compounds **2h**–**k** and **4a**–**d**, respectively. Because ^1^H NMR data for compounds **2h**–**k** and **4a**–**d** have been illegible. ^13^C NMR data are presented for these derivatives.

**2h**. C_20_H_28_N_4_OS (*M* = 372); yield 82.9 %; (δ in ppm; CDCl_3_, 600 MHz); 171.67; 161.18; 159.80; 137.06; 129.94; 128.00; 127.15; 122.37; 59.28; 52.05; 45.42; 43.59; 33.16; 27.08; 20.46; 13.29;. TLC (dichloromethane: methanol: 10:1) R_f_ = 0,36.

IR (for dihydrobromide; KBr) cm^−1^: 3399, 3104, 3077, 2974, 2919, 2793, 2919, 2793, 2703, 2664, 2576, 2465, 1599, 1501, 1439, 1406, 1275, 1218, 1187, 1122, 1072, 1029, 998, 967, 841, 798, 723, 637, 566, 463.

MS *m*/*z* (relative intensity) 372 (M^+^, 17), 274 (66), 261 (13), 152 (17), 139 (41), 126 (24), 111 (17), 105 (100), 77 (33).

**Table:** Elemental analysis for dihydrobromide C_20_H_30_Br_2_N_4_OS (*M* = 534.37)

	C	H	N
Calculated	44.91 %	5.28 %	10.48 %
Found	45.00 %	5.47 %	10.58 %

mp_dihydrobromide_ 227–228 °C

**2i**. C_21_H_30_N_4_OS (*M* = 386); yield 71.9 %; (δ in ppm; CDCl_3_, 600 MHz); 171.53; 161.18; 159.80; 139.83; 133.26; 128.69; 126.73; 121.78; 60.08; 52.05; 46.07; 44.05; 33.09; 28.34; 21.50; 20.46; 13.29;.TLC (dichloromethane: methanol: 10:1) R_f_ = 0.28.

IR (for dihydrobromide; KBr) cm^−1^: 3431, 3102, 3000, 2926, 2768, 2569, 2514, 2462, 1597, 1478, 1455, 1406, 1362, 1291, 1276, 1184, 1122, 1075, 998, 967, 834, 786, 715, 640, 565, 476.

MS *m*/*z* (relative intensity) 386 (M^+^, 12), 288 (43), 152 (13), 139 (22), 126 (15), 119 (100) 111 (14), 98 (20), 91 (30).

**Table:** Elemental analysis for dihydrobromide C_21_H_30_Br_2_N_4_OS (*M* = 547.8)

	C	H	N
Calculated	46.00 %	5.88 %	10.22 %
Found	45.91 %	5.94 %	10.16 %

mp_dihydrobromide_ 210–212 °C

**2j**. C_20_H_27_ClN_4_OS (*M* = 407); yield 49,5 %; (δ in ppm; CDCl_3_, 600 MHz); 171.86; 161.34; 159.80; 136.81; 132.00; 129.73; 127.53; 121.78; 59.73; 51.27; 46.95; 43.56; 31.33; 27.54; 20.46; 13.29; TLC (dichloromethane: methanol: 10:1) R_f_ = 0.38.

IR (for dihydrobromide; KBr) cm^−1^: 3101, 3072, 2967, 2928, 2759, 2706, 2574, 2463, 1617, 1596, 1441, 1408, 1291, 1215, 1186, 1122, 1093, 1073, 1014, 965, 915, 845, 786, 757, 691, 670, 639, 553, 474.

MS *m*/*z* (relative intensity) 406 (M^+^, 10), 308 (37), 152 (15), 141 (23), 139 (100), 126 (19), 111 (18), 98 (25).

**Table:** Elemental analysis for dihydrobromide C_20_H_29_Br_2_ClN_4_OS (*M* = 568.81)

	C	H	N
Calculated	42.22 %	5.14 %	9.85 %
Found	42.33 %	5.01 %	9.98 %

mp_dihydrobromide_ 221–223 °C

**2k**. C_20_H_27_N_5_O_3_S (*M* = 417); yield 75,5 % (δ in ppm; CDCl_3_, 600 MHz); 171.98; 161.57; 159.87 148.38; 143.12; 127.64; 123.71; 121.87; 55.24; 45.42; 43.81; 33.25; 27.89; 20.53; 13.32; TLC (dichloromethane: methanol: 10:1) R_f_ = 0.43.

IR (for dihydrobromide; KBr) cm^−1^: 3430, 3102, 1620, 1597, 1522, 1439, 1410, 1352, 1290, 1179, 1073, 1031, 965, 869, 851, 747, 723, 639, 558, 457.

MS *m*/*z* (relative intensity) 417 (M^+^, 22), 319 (100), 208 (21), 152 (32), 139 (75), 126 (26), 120 (26), 111(31), 104(31), 98 (64).

**Table:** Elemental analysis for dihydrobromide C_20_H_29_Br_2_N_5_O_3_S (*M* = 579.37)

	C	H	N
Calculated	41.46 %	5.05 %	12.09 %
Found	41.45 %	5.07 %	12.05 %

mp_dihydrobromide_ 195–197 °C

**4a**. C_15_H_29_Br_3_N_4_OS (*M* = 372); yield 80,1 %; (δ in ppm; CDCl_3_, 600 MHz); 172.87; 159.28; 138.48; 131.10; 130.04; 128.00; 126.46; 120.54; 56.47; 51.26; 45.44; 39.64; 32.76; 26.28; 20.49; 13.29;.TLC (dichloromethane:methanol: 19:1) R_f_ = 0.32.

IR (for dihydrobromide monohydrate; KBr) cm^−1^: 3509, 3436, 3046, 2971, 2923, 2681, 2586, 2522, 2464, 2084, 1629, 1607, 1575, 1443, 1402, 1360, 1294, 1221, 1098, 1075, 1023, 969, 794, 743, 714, 631, 546.

MS *m*/*z* (relative intensity) 372 (M^+^, 24), 274 (40), 237 (60), 224 (100), 152 (21), 139 (30), 112 (20), 105 (64), 98 (34), 77 (34).

**Table:** Elemental analysis for dihydrobromide monohydrate C_20_H_30_Br_2_N_4_OS H_2_O (*M* = 552.39)

	C	H	N
Calculated	43.48 %	5.84 %	10.14 %
Found	43.73 %	5.74 %	10.20 %

mp_dihydrobromide_ 224–226 °C

**4b**. C_21_H_30_N_4_OS (*M* = 387) yield 79,2 %; (δ in ppm; CDCl_3_, 600 MHz); 172.67; 159.80; 140.06; 138.48; 128.32; 125.97; 120.45; 56.39; 51.34; 45.42; 39.75; 32.84; 26.16; 21.50; 20.46; 13.29; TLC (dichloromethane: methanol: concentrated ammonium hydroxide 89:10:1) R_f_ = 0.51.

IR (for dihydrobromide; KBr) cm^−1^: 3430, 3079, 2967, 2920, 2637, 2564, 2452, 1611, 1479, 1437,1400, 1285, 1270, 1199, 1068, 1039, 968, 925, 873, 839, 757, 726, 583, 508.

MS *m*/*z* (relative intensity) 386 (M^+^, 20), 288 (27), 237 (80), 224 (95), 152 (25), 139 (28), 119 (100)112 (31), 111 (45), 98 (39), 91 (36).

**Table:** Elemental analysis for dihydrobromide C_20_H_30_Br_2_N_4_OS (*M* = 534.37)

Calculated	45.99 %	5.88 %	10.22 %
Found	45.92 %	5.91 %	10.16 %

mp_dihydrobromide_ 196–198 °C

**4c**. C_20_H_27_ClN_4_OS (*M* = 407) yield 78,3 %; (δ in ppm; CDCl_3_, 600 MHz); 172.87; 159.28; 138.53; 136.18 129.26; 128.96; 127.53; 120.00; 56.39; 51.23; 45.57; 39.61; 32.82; 26.25; 20.52; 13.30; TLC (dichloromethane: methanol: concentrated ammonium hydroxide 89:10:1) R_f_ = 0.74

IR (for dihydrobromide; KBr) cm^−1^: 3522, 3422, 3034, 2988; 2938, 2896, 2656, 2569, 2458, 1622, 1430, 1399, 1339, 1291, 1257, 1174, 1089, 1039, 968, 832, 793, 758, 728, 682, 600, 552, 480.

MS *m*/*z* (relative intensity) 406 (M^+^, 18), 288 (27), 308 (28), 237 (34), 224 (100), 152 (64), 141 (21), 139 (92), 112 (31), 111 (43), 98 (45).

**Table:** Elemental analysis for dihydrobromide C_20_H_29_Br_2_ClN_4_OS (*M* = 568.81)

Calculated	42.22 %	5.14 %	9.85 %
Found	42.41 %	5.22 %	9.61 %

mp_dihydrobromide_ 206–208 °C

**4d**. C_20_H_27_N_5_O_3_S (*M* = 417) yield 83.0 %; (^13^C δ in ppm; CDCl_3_, 600 MHz); 172.98; 159.67; 148.27; 140.43; 138.48; 126.87; 123.71; 120.51; 56.42; 51.56; 45.48; 39.81; 32.76; 26.22; 20.51; 13.32; TLC (dichloromethane: methanol: 10:1) R_f_ = 0.43.

IR (for dihydrobromide monohydrate; KBr) cm^−1^: 3451, 3039, 2968, 2934, 2903, 2784, 2696, 2601, 2515, 2457, 1625, 1599, 1524, 1445, 1429, 1404, 1353, 1290, 1260, 1176, 1095, 1033, 1009, 968, 870, 742, 725.

MS *m*/*z* (relative intensity) 417 (M^+^, 26), 319 (55), 237 (20), 224 (100), 152 (27), 150 (39) 141 (21), 139 (34),120 (25), 112 (29), 111 (68), 98 (88).

**Table:** Elemental analysis for dihydrobromide monohydrate C_20_H_29_Br_2_N_5_O_3_S H_2_O (*M* = 597.39)

Calculated	40.20 %	5.23 %	11.72 %
Found	40.46 %	5.03 %	11.77 %

mp_dihydrobromide_ 195–197 °C

### Pharmacology

All compounds were tested for H_3_ antagonistic effects in vitro on the guinea-pig jejunum using standard methods (Vollinga *et* *al*., 1992).

Male guinea pigs weighing 300–400 g were killed by a blow on the head. A portion of the small intestine, 20–50 cm proximal to the ileocaecal valve (jejunum), was removed and placed in Krebs buffer (composition (mM) NaCl 118; KCl 5.6; MgSO_4_ 1.18; CaCl_2_ 2.5; NaH_2_PO_4_ 1.28; NaHCO_3_ 25; glucose 5.5 and indomethacin (1 × 10^−6^ mol/L)). Whole jejunum segments (2 cm) were prepared and mounted between two platinum electrodes (4 mm apart) in 20 mL Krebs buffer, continuously gassed with 95 % O_2_:5 % CO_2_ and maintained at 37 °C. Contractions were recorded isotonically under 1.0 g tension with Hugo Sachs Hebel–Messvorsatz (Tl-2)/HF-modem (Hugo Sachs Electronik, Hugstetten, Germany) connected to a pen recorder. After equilibration for 1 h with every 10 min washings, the muscle segments were stimulated maximally between 15 and 20 V and continuously at a frequency of 0.1 Hz and a duration of 0.5 ms, with rectangular-wave electrical pulses, delivered by a Grass Stimulator S-88 (Grass Instruments Co., Quincy, USA). After 30 min of stimulation, 5 min before adding (R)-α-methylhistamine, pyrilamine (1 × 10^−5^ mol/L concentration in organ bath) was added, and then cumulative concentration–response curves (half-log increments) of (R)-α-methylhistamine, H_3_-agonist were recorded until no further change in response was found. Five minutes before adding the tested compounds, the pyrilamine (1 × 10^−5^ mol/L concentration in organ bath) was added, and after 20 min cumulative concentration–response curves (half-log increments) of (R)-α-methylhistamine, H_3_-agonist, were recorded until no further change in response was found. Statistical analysis was carried out with the Students’ *t* test. In all tests, *p* < 0.05 was considered statistically significant. The potency of an antagonist is expressed by its pA_2_ value calculated from the Schild (Arunlakshana and Schild, 1959) regression analysis where at least three concentrations were used. The pA_2_ values were compared with the potency of thioperamide.
